# Remote Inclusion of Vulnerable Users in mHealth Intervention Design: Retrospective Case Analysis

**DOI:** 10.2196/55548

**Published:** 2024-06-14

**Authors:** Ingjerd J Straand, Kimberley A Baxter, Asbjørn Følstad

**Affiliations:** 1 Department of Social Work University of Stavanger Stavanger Norway; 2 School of Exercise and Nutrition Sciences Queensland University of Technology Brisbane Australia; 3 Centre for Childhood Nutrition Research Faculty of Health Queensland University of Technology Brisbane Australia; 4 Department of Sustainable Communication Technologies Sintef Digital Oslo Norway

**Keywords:** user testing, user participation in research, COVID-19, remote testing, intervention design, mobile phone

## Abstract

**Background:**

Mobile health (mHealth) interventions that promote healthy behaviors or mindsets are a promising avenue to reach vulnerable or at-risk groups. In designing such mHealth interventions, authentic representation of intended participants is essential. The COVID-19 pandemic served as a catalyst for innovation in remote user-centered research methods. The capability of such research methods to effectively engage with vulnerable participants requires inquiry into practice to determine the suitability and appropriateness of these methods.

**Objective:**

In this study, we aimed to explore opportunities and considerations that emerged from involving vulnerable user groups remotely when designing mHealth interventions. Implications and recommendations are presented for researchers and practitioners conducting remote user-centered research with vulnerable populations.

**Methods:**

Remote user-centered research practices from 2 projects involving vulnerable populations in Norway and Australia were examined retrospectively using visual mapping and a reflection-on-action approach. The projects engaged low-income and unemployed groups during the COVID-19 pandemic in user-based evaluation and testing of interactive, web-based mHealth interventions.

**Results:**

Opportunities and considerations were identified as (1) reduced barriers to research inclusion; (2) digital literacy transition; (3) contextualized insights: a window into people’s lives; (4) seamless enactment of roles; and (5) increased flexibility for researchers and participants.

**Conclusions:**

Our findings support the capability and suitability of remote user methods to engage with users from vulnerable groups. Remote methods facilitate recruitment, ease the burden of research participation, level out power imbalances, and provide a rich and relevant environment for user-centered evaluation of mHealth interventions. There is a potential for a much more agile research practice. Future research should consider the privacy impacts of increased access to participants’ environment via webcams and screen share and how technology mediates participants’ action in terms of privacy. The development of support procedures and tools for remote testing of mHealth apps with user participants will be crucial to capitalize on efficiency gains and better protect participants’ privacy.

## Introduction

Mobile health (mHealth) interventions, which use mobile technology such as smartphone apps to promote healthy behaviors or mindsets [1], are a promising avenue to reach vulnerable groups [2]. Meaningful user involvement is critical for such interventions [3] to ensure that end user needs and perspectives are adequately represented in the design process [4]. Conducting such feedback and evaluations with users face to face (local testing) [5-7] involves efficiency drawbacks, particularly travel, time, and cost [8]. Researchers and practitioners have thus experimented with remote testing and research [9,10] using both specialized tools (eg, UserTesting and Lookback) and videoconferencing (eg, Zoom, Hangout, and Teams). Studies comparing local and remote research practices have concluded comparable results in the quality of the research output [11]. However, before the COVID-19 pandemic, local testing was the usual practice in research and among practitioners [4,6]. Reasons may include network variance, poor audio or video quality, unfamiliarity with remote technology, and the lack of contextual information or nonverbal cues inherent in remote methods. Local testing, by contrast, removes users from the intended context of use; this is significant for user involvement in the design of mobile solutions such as mHealth interventions.

Traditional research methods tend to involve users from high socioeconomic backgrounds, who are easy to reach and have the means to participate, including resources of time, transport, and social support [12]. Human-computer interaction research calls for adequate reach and engagement with the people affected by the design to ensure an alignment of needs and, ultimately, an effective program [13,14]. This can be challenging when working with community groups who are marginalized or experience social disadvantage, such as racial or ethnic minority groups, individuals who have low income and who are unemployed, people with disabilities [15], or those with gender or sexual diversity [14,16]. This risks diminishing the validity of the findings to the target population and reduces the authenticity of engagement. While mHealth interventions may be particularly relevant for these groups, the suitability of remote practices for user involvement should be explored. More evidence is needed to support the appropriateness and effectiveness of remote user-centered research methods when engaging with vulnerable participants.

Accelerated by the pandemic, remote research and participation tools have become more available and ready-to-hand [17]. Researchers expedited the incorporation of remote methods that allowed for project continuity, highlighting the research community’s resilience and researchers’ and participants’ willingness to experiment with technology. A recent meta-analysis found that one of the most significant effects of the pandemic on user involvement in design was shifting to web-based platforms [11]. At the community level, the increased use of telehealth services across populations to provide continuity of health care and education [18] has increased familiarity and comfort with videoconferencing and other web-based tools.

While the COVID-19 pandemic was a catalyst for innovation and creativity in remote user design methods, now that the pandemic has resolved [19], the opportunity to learn and adopt effective remote methods remains. Conducting meta-research to capture these experiences is important for future research applications. Some examples of such research exist: Hill et al [20] reviewed practical approaches for remote user testing in older adults. Other researchers have compared findings between remote and nonremote methods [21] or discussed specific aspects of the testing, including moderator and observer roles [5,22]. However, few studies have detailed the implementation of user-centered design in mHealth [3] or reflected on the researcher and participant experiences [23] in intervention design targeting vulnerable or diverse population groups.

Thus, the research question addressed by this qualitative and retrospective study is as follows: what are the opportunities and considerations emerging from involving vulnerable user groups remotely in mHealth intervention design? This study will highlight what was learned by adapting to agile remote user involvement during COVID-19 to inform future applications of such involvement with vulnerable user groups. Research practices from 2 projects, which applied remote inclusion of vulnerable population groups to designing and developing mHealth interventions within child health (parental feeding) and social psychology (mindset), were used as cases.

This study is structured as follows: an overview of the research projects and the methodology of this study is provided, followed by case descriptions and lessons learned before the analytical findings and implications are presented.

## Methods

### Research Context

The 2 research projects in this study used human-centered design (HCD) methodology [24] to design and develop web-based mHealth interventions targeting vulnerable populations. The project aims were to create digital health interventions collaboratively with and for end users and then evaluate these as part of ongoing research. The Responsive Feeding in Tough Times (RFiTT) project in Australia aimed to develop and evaluate a parenting program to promote responsive feeding practices in parents with young children in low-income families. The Career Learning App (CL-APP) project in Norway aimed to design, develop, and test positive psychology intervention apps targeting unemployed adolescents and young adults to promote job-seeking mindset and behaviors. These projects from different contexts have shared characteristics, including transdisciplinary work across design and health and applying an HCD process where users’ ideas and feedback were central to the final intervention designs. Both project outcomes were web-based interventions designed for self-administered use on users’ mobile phones, and remote user testing was applied with research participants from vulnerable groups.

In the 2 projects, the respective authors (IJS and KAB) developed user-centered design approaches, which were predominantly formative user-based evaluation [4,6] in the form of qualitative, moderated early testing [25] and feedback on intervention prototypes. This included conducting the posttest analysis of the collected data. From March 2020 to December 2022, a total of 38 sessions were conducted across the 2 research projects. Participants were recruited intentionally with the characteristics of potential end users of the interventions to include their input into the designed outcome.

The projects’ remote user engagement timing aligned with different phases of the COVID-19 pandemic. The Norway project experienced acute disruption during user testing (March 2020-April 2021), coinciding with the initial COVID-19 response. In contrast, the Australian project conducted user testing (November 2022-December 2022) during a more stable “living with” COVID-19 phase.

### Research Design

Given the unprecedented COVID-19 pandemic during user testing, the retrospective reflection-on-action approach [26,27] was selected to explore the remote research setting. This study’s research question and topic were explored [28,29] through “reflecting on action” [26]. Reflection was both internal and in dialogue between the authors and fellow researchers. This approach enabled researchers to reflect on the cases after the upheaval period of the pandemic had receded to uncover knowledge through analyzing and integrating experiences and practices.

We conducted a descriptive and retrospective examination of the research practices and experiences across the 2 projects. This was done through an iterative process using visual mapping (ie, affinity mapping or KJ-method) to sort findings visually [30] in Miro [31]. Affinity mapping builds upon abductive thinking and is commonly used by user experience practitioners [32,33]. This method was selected because of our heterogeneous data set [32] and the need to synthesize ideas from unstructured data. Our data included multiple sources: protocol documents, user test setups and documentation, researcher notes and reflections, postanalysis reports, and photos and screenshots from recordings.

**Figure 1:**
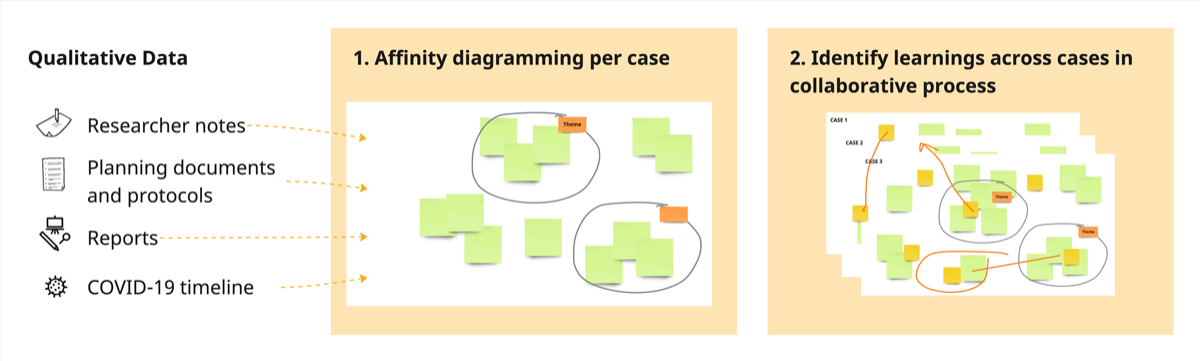
Stepwise analysis process based on visual diagramming, such as affinity mapping.

We took a constructivist approach to our analysis, where synthesis and connections are formed through the researchers’ critical reflection, and learnings are identified through active engagement and “discussions with the data” [34,35]. Our analysis was conducted stepwise (Figure 1), where we first added our data to the diagrams and started making clusters and groupings of findings relevant to the research question and labeling these on a case-by-case basis. Second, we identified learnings across cases in a collaborative process by regrouping our initial categories and findings of interest into broader categories or constructed themes [34] that represent the opportunities and considerations from different cases.

### Participants

The participants included in this study are considered potentially “vulnerable” due to socioeconomic factors such as unemployment, low income, and economic hardship. *Vulnerability* is viewed as an inclusive term in line with the study by Culén and van der Velden [36], assuming that all users may be “vulnerable” at some point. The 2 research projects had different participant groups and ethical considerations; therefore, we describe them separately below. Table 1 summarizes participants across projects.

**Table 1:** Description of participants across cases: demography, recruitment channel, and format of the user testing.

		Participants
**Case 1**		
	Target group (inclusion criteria)	Unemployed or dropped out of school Aged 18-29 y Living in Rogaland, Norway Speaking Norwegian
	Recruitment channel	Invitation via NAV^a^ or IPS^b^ program Self-signup on website or via SMS text messaging or mail
	Participants	12 participants aged 18-27 y; 7 females, 5 males^c^; 2 from ethnic minority groups (immigrant or BIPOC^d^)
	Format user test	3 in-person sessions 9 remote of which 3 Discord, 5 Zoom, 1 other (Whereby); 2 used mobile phone device to connect to Zoom
**Case 2**		
	Target group (inclusion criteria)	Unemployed or dropped out of school Aged 18-29 y Living in Rogaland, Norway Speaking Norwegian
	Recruitment channel	Invitation via NAV or IPS program Self-signup on website or via SMS text messaging or email
	Participants	13 participants Aged 18-29 y; 6 female, 7 male^c^; 1 ethnic minority group (immigrant or BIPOC)
	Format user test	0 in person (not possible) 13 remote of which 12 Zoom, 1 other (Teams), and 1 used a mobile device to connect to Zoom but switched to computer during session
**Case 3**		
	Target group (inclusion criteria)	Parent or caregiver of a child aged 6 mo-3 y Aged >18 y Self-reported economic hardship
	Recruitment channel	Expression of interest list
	Number of participants	12 participants Average age 30 (range 26-36) y; 10 female, 2 male; 9 Australian, 1 Aboriginal Torres Strait Islander, 1 Indian, 1 Cambodian
	Format user test	12 Zoom 10 used mobile phone devices to connect to Zoom

^a^NAV: Norwegian Labour and Welfare Administration.

^b^IPS: Individual Placement and Support program.

^c^On the basis of observation, not self-reported.

^d^BIPOC: Black, indigenous, and people of color.

### Ethical Considerations

#### Norway: Participants, Ethical Considerations, and Approval

Participants recruited to the Norwegian project were 18 to 29 years old and either registered as unemployed at the Norwegian Labour and Welfare Administration (NAV) or participating in a regional Individual Placement and Support program. Furthermore, they needed to speak Norwegian because of the in-app language. All participants provided explicit and written consent to participate in the study and were compensated for their time with a gift card of US $30 per session. The study was evaluated and approved by the Norwegian Centre for Research Data (approval number 131074).

#### Australia: Participants, Ethical Considerations, and Approval

Participants were recruited Australia-wide and self-identified as experiencing economic hardship during screening. All participants were aged >18 years and caregivers of a child between 6 months and 3 years of age. Individuals were recruited from a pool of potential participants who had previously taken part in a web-based survey and had expressed interest in being contacted about other research activities. Participants were given an electronic gift voucher worth US $18 to thank them for their time. The Children’s Health Queensland Hospital and Health Service Human Research Ethics Committee (LNR/21/QCHQ/72314) and the Queensland University of Technology Human Research Ethics Committee (2021000193) approved the study.

### Case Descriptions

**Figure 2:**
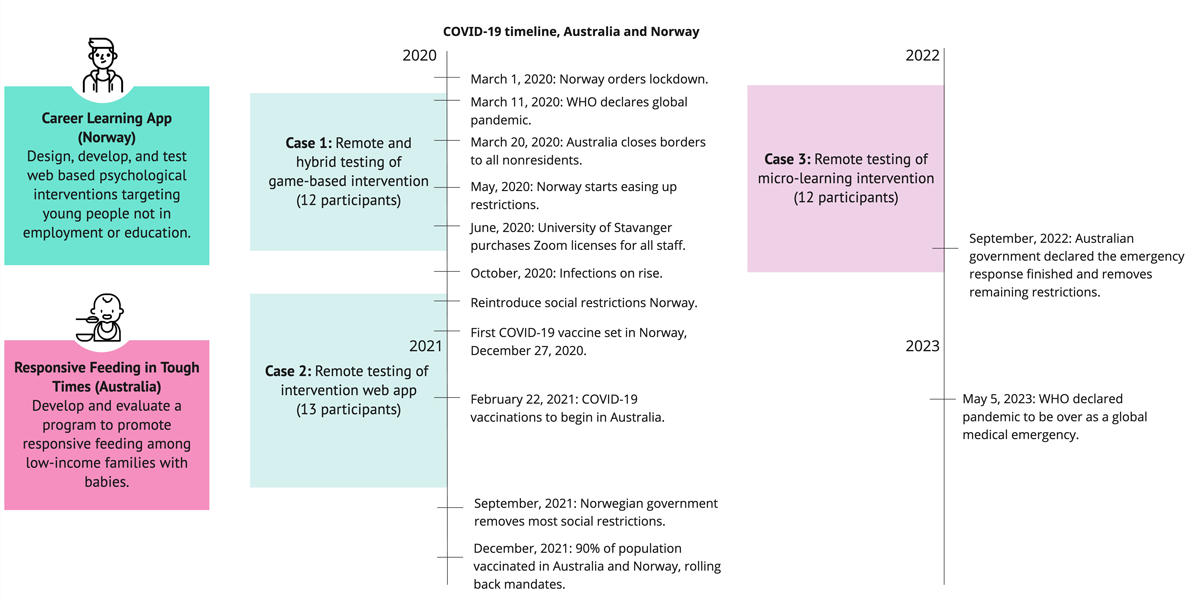
Overview of user involvement conducted in the 2 research projects during the pandemic.

This section outlines the 2 research projects and details the user involvement protocols. The Norwegian project included 2 instances of user involvement; the Australian project involved 1. Hence, 3 cases are presented across the 2 research projects (Figure 2). Each case is divided into *case description*, *pandemic restrictions*, *test setup*, *participants*, and *case-specific reflections.*

#### Case 1: Remote and Hybrid Testing of a Game-Based Intervention Concept, Norway

##### Case Description

**Figure 3:**
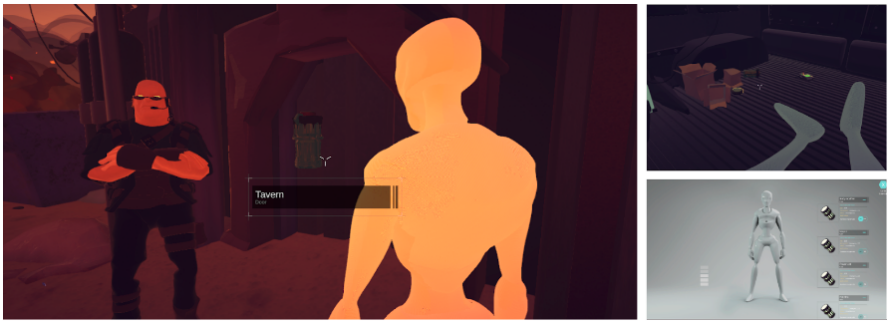
Screenshots from 3D game intervention VitaNova.

The CL-APP project explored an interactive gaming concept to make a positive psychology intervention more engaging and relevant to unemployed young adults. The intervention design explored a 3D-based game. The development work was planned and executed in 3 sprints, with end users involved in formative user testing toward the end of each sprint. Further elaboration of the game concept and user feedback can be found in the study by Straand et al [37], with screenshots provided in Figure 3.

##### Pandemic Situation

On March 12, 2020, the Norwegian government ordered a nationwide lockdown, closing nonessential workplaces, schools, and child care centers. Schools and child care reopened with reduced hours for younger children toward the end of the following month. In May 2020, social and mobility restrictions were eased or replaced with mask mandates and sanitation requirements. However, in September 2020 and October 2020, infections again peaked, and in late October, new restrictions were announced, returning nonessential workers such as university staff to home offices.

##### Participants

A total of 12 participants (female participants: 7/12, 58%; male participants: 5/12, 42%), aged 18 to 27 years, participated in the study.

##### Test Setup

In mid-March 2020, amidst pandemic uncertainty and lacking established remote protocols, plans for moderated, in-person usability testing were improvised. Discord, chosen for its familiarity among young gamers, served as the platform for remote testing. The test setup involved several manual operations due to the lack of functionality in Discord, including scheduling, consent, and provision of gift cards. The moderator and observer met a few minutes before and then added the participant to a group call once the participant had logged in to Discord. Despite its suitability for gamers and developers, approximately half of the users encountered startup issues due to unfamiliarity with the software. External software (Apple QuickTime) was used for recording, and this lack of a built-in recorder led to missing audio for some sessions. Platforms designed for usability testing or videoconferencing were rejected at the time from the premise of introducing complexity for the team and the participant users for a relatively short time of need.

**Figure 4:**
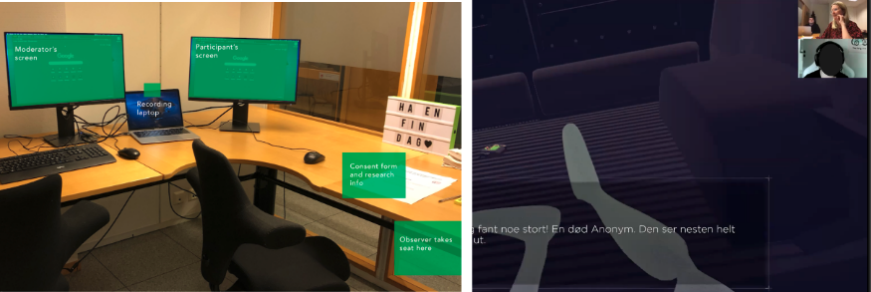
Hybrid test setup: a total of 2 participants used local testing (left), while the remaining users opted for Zoom meetings (right).

As the team transitioned toward testing a functional prototype, concerns over network variance and load time prompted plans for face-to-face testing once restrictions eased in May 2020. A single participant signed up who had been involved in early-stage interviews. The test was conducted with strict sanitation and social distancing. However, with only 1 participant, it had limited value. A subsequent round of testing was planned for October 2020, when COVID-19 restrictions were expected to ease. This time, participants self-enrolled via a website and received SMS text messaging confirmation and reminders. We set up a testing space within the NAV offices. The team adapted its research strategy to allow participants to choose between in-person and remote testing on Zoom on the enrollment website. Remote participants signed digital consent forms and received digital gift cards, while in-person participants completed forms upon arrival and received physical gift cards (see Figure 4 for the hybrid test setup). The team completed tests with 7 participants, with the majority (5/7, 70%) opting for Zoom sessions.

##### Case-Specific Reflections

This case involved improvisation to enable continuity of the research, both with software and tools and with testing procedures. This iteration allowed us to observe how the videoconferencing software impacted the interaction with the participant, creating a new setting for the interaction depending on the software used. In the first rounds of testing using Discord, we all had our camera off. Discord users mostly use illustrations or avatars for their profile pictures and audio-only calls. Thus, although it is possible to share a camera view, none of the sessions using Discord had the participants with camera on; this included our webcams as researchers in the role of moderator and observers. The sessions done via videoconferencing software always had the camera-on mode for the moderator and nearly always for the participants, offering a richer data set for later analysis.

The “hybrid” strategy toward the end of the study meant the moderator and observer were usually in the same room, calling in as 1 user on Zoom. After the first session, it became the established practice for the moderator and observer to join in as individual users; the observer would mute the camera and microphone after a brief introduction at the beginning. This improved the interaction of the session, as the participant did not have to address 2 people. This remote setup allowed the observer to “disappear” into the background, overcoming the issue with the silent notetaker in a face-to-face session.

Some tasks were more challenging to deliver in the remote setup since the test tasks were designed for in-person sessions rather than remote participation. For instance, idea cards were created that participants could sort according to their preferences. When the testing was on the web, we had to send them a copy of the cards in PDF format; this made the task less engaging and cumbersome. After preliminary user-derived findings, the development of the gaming-based intervention app presented in case 1 was discontinued.

#### Case 2: Remote Testing of mHealth Intervention Web App Concept, Norway

##### Case Description

**Figure 5:**
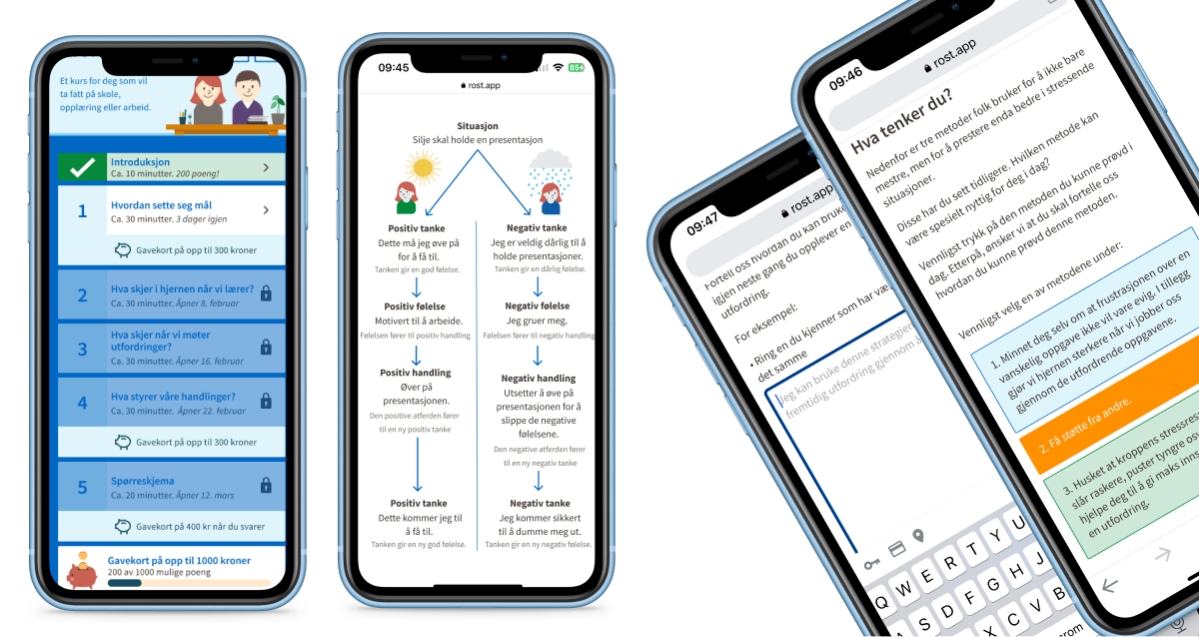
Intervention web app concept shaped as learning modules.

Building on case 1, the CL-APP project redirected the design and development process to a mobile phone web app based on user preferences. The intervention target was foremost to promote a “growth mindset.” A growth mindset [38-40] encourages a different interpretation of challenges faced by the young unemployed, normalizing struggles and setbacks to offer a more positive and flexible view of one’s intelligence and ability to learn new things. The key objective of engaging with end users was to explore users’ motivation to enhance reach and adherence. HCD methods ensured that the intervention was relevant, user-friendly, and motivating (see Figure 5 for screenshots of the app). In this process, researchers collaborated with designers, developers, and stakeholders, including end users, from October 2020 until the app’s completion in December 2022. The user testing took place between November 2020 and April 2021.

##### Pandemic Situation

On October 26, 2020, the Norwegian government announced new health restrictions to reduce social interaction at work and home, strongly recommending that people return to home offices where possible [41].

##### Participants

Participants were recruited for 4 rounds of testing. A total of 13 participants (female participants: 6/13, 46%; male participants: 7/13, 54%) aged 18 to 29 years participated in the study.

##### Test Setup

Given the work-from-home directive at the time, the design process, including interaction with end users, was planned remotely via Zoom. During the design process, 4 rounds of testing were performed: the initial test to understand what should be altered in the existing intervention (November: 4 participants) and 3 instances to get feedback on new designs with increasing levels of fidelity as the design progressed (January: 5 participants, February: 2 participants, and March-April: 4 participants). Prototypes were tested using the design tool Figma. The sessions were completed at times that were suitable to the participant. There was 1 session in the evening, but most participants opted for midday sessions (around 11 AM-2 PM).

##### Case-Specific Reflections

**Figure 6:**
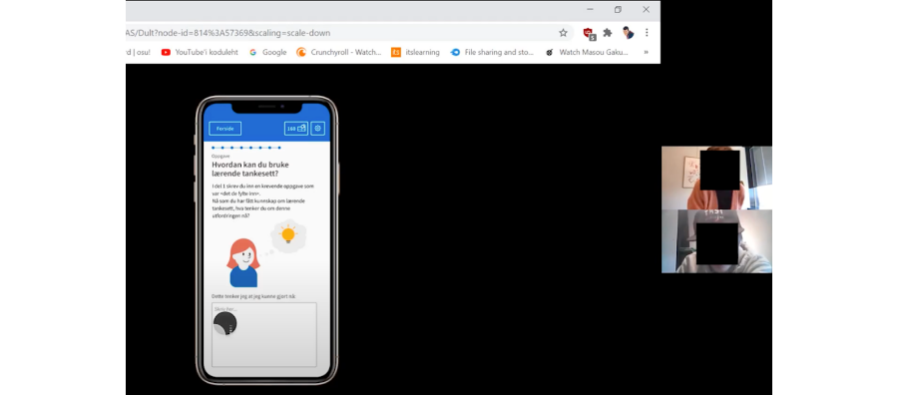
Remote test setup in Zoom. The moderator has the camera on; the observer has the camera and microphone muted, therefore hidden from view.

Our main challenge was that the prototype was designed for mobile use, and screen sharing from devices was troublesome in Zoom. Thus, for most of the tests on the new designs, we relied on desktop use and screen sharing from the browser (Figure 6). When a participant dialed in from their phone, the prototype view became unreadable, and we had to ask the participant to switch over to a device with a larger screen. All participants and moderators had their cameras switched on (unprompted). After an initial round of introductions, we continued switching the camera off for the observer or notetaker to reduce their presence in the user-researcher interaction.

#### Case 3: Remote Testing of an mHealth Microlearning Concept−Australia

##### Case Description

The RFiTT research program aimed to develop and evaluate an intervention to promote optimal child-feeding practices among low-income families. The secondary aims were to determine the feasibility, satisfaction, and acceptability of the mode of delivery. Families experiencing socioeconomic disadvantages face challenges feeding their children and following optimal feeding guidelines. The early years are crucial for establishing optimal feeding practices among parents and developing healthy child eating behaviors [42]. Therefore, the target of the intervention was parents or caregivers of children aged 6 to 24 months.

An mHealth digital microlearning concept was developed in response to parent engagement during the project’s development phase [43]. Project constraints dictated a technology platform that required no software engineering or development phase and could be generated within a 4- to 6-month time frame. Web-based no-code technologies were researched and piloted to determine a suitable platform.

**Figure 7:**
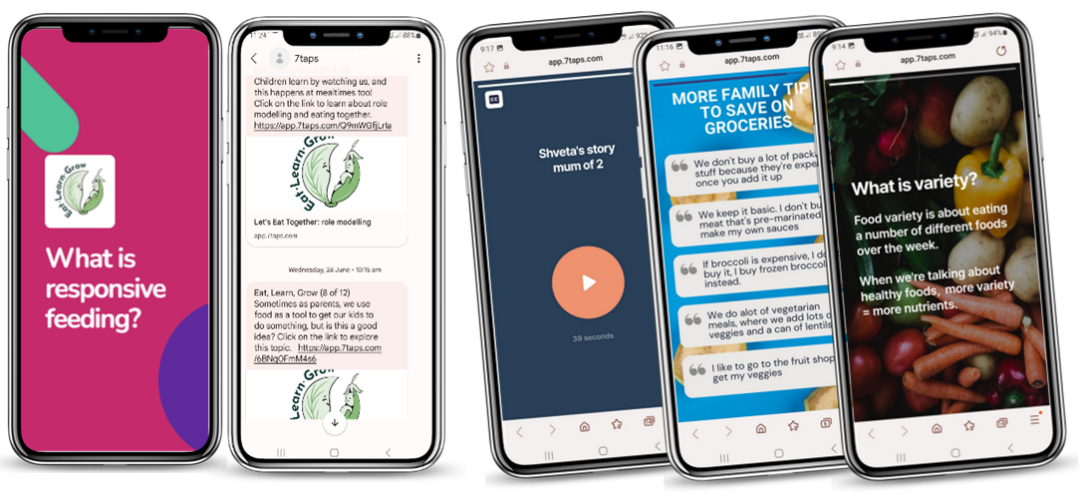
Eat, Learn, Grow intervention: examples of digital module content and SMS text messaging delivery system.

A learning technology platform (7taps), which used microlearning education, was selected. This platform enabled researchers to create contents that included videos, images, text, and interactions without external input from software engineers or app developers. This platform had a mobile-first design and learning management capability where modules could be delivered with preset timing in customized SMS text messages. Functional prototypes could be created and tested with users using this platform with little to no moderation. A total of 3 test modules that would form part of a microlearning responsive feeding parenting intervention were created (Figure 7).

##### Pandemic Situation

The RFiTT research program commenced in April 2020. The first case of COVID-19 was confirmed in Australia on January 25, 2020 [43]. On March 18, 2020, the federal government declared a biosecurity emergency, and all Australian States and Territories subsequently implemented lockdown measures [44]. Australia only fully opened its international borders to visitors in February 2022.

During the data collection and engagement phase of RFiTT (2021-2022), recruitment was impacted by the COVID-19 pandemic, and face-to-face data collection attempts were challenging. These recruitment challenges led to experimentation with remote research methods (telephone, web-based survey, noncontact equipment drop-offs) for research activities. By the time of the user testing sessions (November to December 2022), the RFiTT research program had adopted a complete remote research methods approach, and the scope of the population target for the intervention had shifted from a local context (Brisbane, Queensland) to Australia-wide.

##### Participants

A total of 12 participants tested the prototypes. Of the group, 42% (5/12) had a university degree, and 3 individuals expressed that they had neurodiversity, which impacted their ability to learn and process information (attention-deficit/hyperactivity disorder, dyslexia, and aphantasia). Further details are available in Table 1.

##### Test Setup

Potential participants were telephoned to invite them to participate in the user testing sessions. Interested participants were sent a digital web link to the Participant Information Statement, a web-based consent form, and a short demographic survey. The web-based form and survey were hosted on REDCap (Research Electronic Data Capture; Vanderbilt University), a secure web application for building and managing web-based surveys [45]. The aim of the testing was twofold: (1) to test the acceptability, readability, and accessibility of 3 examples of microlearning content and (2) to co-design aspects of the content and structure of the intervention.

**Figure 8:**
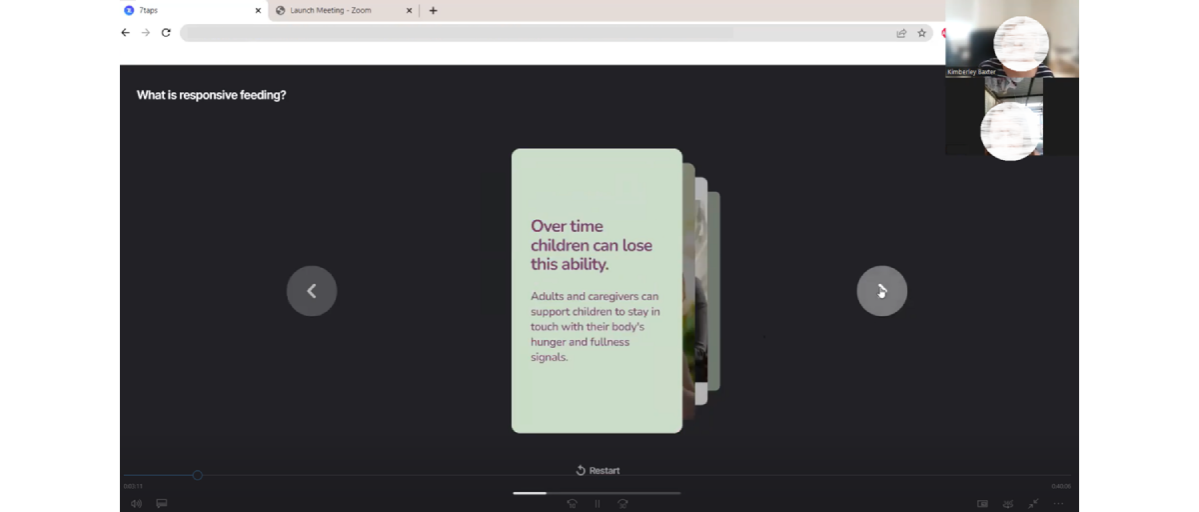
Screenshot of a remote user testing session conducted on Zoom.

The sessions were completed at times suitable to the participant, including out-of-hour sessions from November 7, 2022, to December 1, 2022. Most participants joined the session using their mobile phones (10/12, 83%). A total of 3 modules were designed to present different styles of videos, content, and imagery to elicit feedback on the different formats and parents’ preferences. The module web links were sent via mobile phone SMS text messaging to participants on the day of the arranged session. Parents viewed the content unmoderated. A Zoom session with the lead researcher (KAB) was arranged on the same day to capture parents’ impressions and feedback. All Zoom sessions were video and audio recorded. During the sessions, the researcher shared a preview screen of the digital modules and guided the parent through a talk-out-loud walkthrough of the content (Figure 8). Open-ended questions regarding the usability, accessibility, and satisfaction of the modules were asked. Perspectives from parents were sought on recruitment and retention strategies, the language of key intervention messages, structure, and program timing. The parent intervention was renamed to “*Eat, Learn, Grow*” to reflect parents’ feedback.

##### Case-Specific Reflections

Remote inclusion of participants allowed for representation across Australia and of employed parents, who indicated that they would not have been able to participate if the session had been in person. The Zoom platform was effective; no participants had difficulty downloading or using the software. Most of the group (10/12, 83%) used a mobile phone. The mobile phone screen size restricted viewing the content via screen share (see Figure 8). However, it was acceptable, as participants had just engaged with the content. There were minimal connectivity or audio difficulties, but given the participants’ home environment, there were interruptions from young children being supervised during sessions. These disruptions did not reduce the effectiveness of the sessions and are a common occurrence in research with parents, where young children need to accompany parents. The researcher (KAB) is experienced with children and conducting research with families.

The most significant downside noted for the remote sessions in this case was that the parents engaged with the digital modules unmoderated; therefore, researchers did not observe parents interacting with the content for the first time. In the remote user setup, moderated sessions of parents viewing the content on their mobile device were not possible, given that the device needed to be used for videoconference for the feedback session. Moderating the session may have provided helpful information about parents’ responses to the digital content. However, given that the platform used (7taps) was a purpose-built learning technology designed for first-time users, this was not the aim of the user testing sessions. This was mitigated by conducting the follow-up Zoom session the same day the digital content was sent to parents.

## Results

### Overview

We identified opportunities and considerations of conducting remote research with vulnerable users by reflecting on action and through visual diagramming across cases. These are represented as *reduced barriers to research inclusion, digital literacy transition, contextualized insights: a window into people’s lives, seamless enactment of roles,* and *increased flexibility for researchers and participants.*

### Reduced Barriers to Research Inclusion

During the user testing, the need to quickly design procedures for remote and hybrid research was necessitated by the evolving COVID-19 pandemic. Across the 3 cases, we found that remote research methods effectively engaged the targeted population groups of unemployed young adults and parents experiencing economic hardship. Enabling participants to participate in their home environment removed some systemic barriers to engaging in traditional research. For both groups, there were barriers to meeting face to face beyond the practicalities of travel, time, and capacity. Young people and parent participants displayed increased comfort with digital technologies and remote interactions, facilitating their participation in these research programs.

Remote and agile research methods enabled a broader and more diverse participant pool. RFiTT (case 3) widened the recruitment pool to Australia-wide rather than a small geographical area focus. In the Norway project, the recruitment pool was not widened geographically. However, remote methods enabled continued research during the acute response phase of COVID-19. Toward the end of our testing of the gaming concept (case 1), in-person participation was planned since restrictions had been lifted. However, recruitment was difficult, and participants who did consent failed to attend booked appointments despite a monetary incentive. Through this recruitment period, the population group expressed a high concern about the pandemic to researchers. This experience was confirmed in discussions with stakeholders such as welfare administration staff. Shifting to remote testing via web-conferencing (Zoom) facilitated continued participation.

Remote methods mitigated accessibility barriers and eased participants’ potential fear, whether related to the pandemic or the unknown of being involved in a research project. Furthermore, many tests were conducted in the evening to adapt to the needs of parent participants (case 3). Across our populations, catering for continued remote participation was relevant even after restrictions were relaxed and was demonstrated by participants’ strong preference for remote methods.

### Digital Literacy Transition

Initially, the tools used for remote research were improvised, and methodological planning took an iterative approach. As the pandemic unfolded, users and researchers gained experience with relevant digital technology, reaching greater technology awareness and control. The different time frames in which the case studies were conducted during the COVID-19 pandemic provided a context to explore this trend of what we may refer to as a transition to digital literacy.

Initially, researchers and project stakeholders were reluctant to transition to remote participation (cases 1 and 2), whereas users seemed to prefer remote modes. The preferences of researchers and project stakeholders partly grew out of a desire to conduct the research “as planned” and to use established methods. There was also uncertainty about whether users had the necessary skills to use videoconferencing. Researchers had concerns about the limited opportunity for rapport building through informal conversation before the session started. However, the research team underestimated how digitally literate the participants were. This is unsurprising given the amount of time spent on the internet and the degree of web-based communication and collaboration in both groups across many aspects of life [46,47]. This was coupled with COVID-19 pandemic–driven increases in the use of technology for communication and services, such as telehealth [48-51] and work-from-home needs [52,53].

### Contextualized Insights: A Window Into People’s Lives

Despite our target participants’ familiarity with web-based communication, the rapid adoption of these technologies also required sensitivity in protecting participants’ privacy. Contrary to our perception of poorer conversations with the loss of face-to-face conversation, we experienced *more* entry into users’ lives than participation at a research site. The recording was done easily as a part of the natural flow of conversation with the participant on the web in Zoom. In contrast, introducing video recording devices into physical meetings is cumbersome and can make people uncomfortable. Furthermore, it was found that the type of software used either increased or reduced the likelihood of data sharing due to its internal logics, customs, or *vibe* [54]. With Discord, it is not customary to use a real profile photo; in most instances, people use an avatar, and it did not feel natural to turn the webcam on. Therefore, this channel collected much less personal information than Zoom. Zoom encourages turning webcams on and recording seamlessly and unobtrusively. The tools used for supporting the research, such as Discord and Zoom and systems for issuing electronic gift cards, required collecting more personal data (such as name, email, phone number, and usernames) than in-person research methods.

Web-based and remote methods were a more natural and relevant environment for the user, revealing more contextual information than expected and providing a temporal window into people’s lives. Sometimes, this may include unintended information, such as username, browsing history, or open tabs when participants were screen sharing. The less professional nature of the Zoom session also meant some participants were less formal. In one instance, a participant wore a bath robe, while others had babies crying in the background, pets, or others who entered the conversation. This provided a richer contextual backdrop to who the participants were and sparked informal conversation and trust building. At the same time, this contextualized information from the user tests does introduce privacy concerns.

### Seamless Enactment of Roles

It is sometimes necessary to have observers during user testing. For face-to-face sessions, 2-way mirrors or screencasting to another location may be used to enable observation. Additional observers may also be needed in a physical space to take notes; this can be disruptive. The user may feel uncomfortable talking to 2 people, not knowing who to look at when talking and when someone is writing intensively. Remote user sessions may require fewer observers, and they may be less intrusive when they are present.

In the Norway cases, the observer’s role as a notetaker was improved by videoconferencing. The observer and interviewer would have the camera on for the start of the testing. Then, after introductions, the observer could mute the camera and microphone and continue taking notes without impacting the session. If the observer wanted to ask follow-up questions, it was easy and natural to either bring the observer back into the conversation or allow the observer to post questions via a chat channel for the interviewer to follow up. With this more silent observer role, there was little disturbance to the flow of the conversation. It was easy and natural to switch roles during the session, which was done in case 2, where the author (IJS) moderated most of the session, and one of the designers ran through the prototype with the participants. In the Australian case, no person other than the author (KAB) was present for the testing.

### Increased Flexibility for Researchers and Participants

Remote-only testing was found to be more streamlined and flexible compared with both in-person and hybrid models. Research participation, which is planned to be hybrid (case 1), requires booking and setting up the room. This introduces limitations on the remote research imposed by the physical meetings, such as the timeline and availability of physical space.

Remote testing (cases 2 and 3) allowed for more flexibility; meetings could be conducted in the evenings or during weekends or holidays to accommodate participants, with minor disruption to researchers who could dial in from home but with great benefits to participants. Without the booking and timeline constraints of physical space, sessions could take place over time (case 2), allowing revision and adaptation of design prototypes that could be tested again. This maximized the data collection capacity of the sessions and led to a more agile approach to our research and engagement with participants.

For RFiTT (case 3), most participants (10/12, 83%) engaged with the remote user testing sessions via mobile phone. Participants did not have access to a working computer, and using a mobile device enabled participants to perform essential tasks such as supervising young children. This suggests that flexibility and convenience to do other things may contribute to the preference for remote participation. For CL-APP (cases 1 and 2), nearly all participants connected to the remote testing sessions on their computers (23/25, 92%). Participants had good access to both computers and smartphones. The preference for remote participation in this project was considered to be convenience factors, social anxiety, and COVID-19–related concerns. Further research is needed to verify the reasons for preferring digital and remote engagement with research across different populations.

## Discussion

### Principal Findings

The global pandemic necessitated the reevaluation of traditional research methodologies, compelling researchers across disciplines to adapt to the changing environment and adopt agile approaches. This study explored opportunities and considerations from involving vulnerable user groups remotely to provide lessons learned for future research. We did this by reflecting on research practices that involved user-centric evaluation of interactive behavioral and psychological intervention designs. A total of five topics emerged from our analysis: (1) reduced barriers to research inclusion; (2) digital literacy transition; (3) contextualized insights: a window into people’s lives; (4) seamless enactment of roles; and (5) increased flexibility for researchers and participants.

Across the 3 cases, remote participation contributed to a more accessible inclusion of users in design. The emerging technology on modern mobile phones offers the potential to engage with participants effectively across digital platforms such as Zoom. Mobile smartphones are prolific, and with the declining cost of data [55], remote methods that seamlessly integrate with mobile devices are becoming more accessible and equitable for user engagement. Low-income user groups may have limited access to working laptops or home computers, as was the situation in case 3 of this study. Adequate provision or access to suitable digital devices is important in digital equity and research in vulnerable groups [56].

Remote methods mitigate accessibility barriers such as travel costs and logistical challenges, which may deter participation from vulnerable groups. In countries such as Australia and Norway, with a diverse and “spread out” geographical landscape, this was highly valuable in the intervention development phase, enabling wider recruitment reach. This also has significant implications for scalability and implementation. A broader recruitment scope may make it easier to include more participants who are less represented in research, such as those living in rural areas [14]. Web-based and remote methods of research engagement, such as social media, may facilitate engagement with vulnerable groups not connected with organizations, workplaces, or other services [12].

With an increased focus on digital health interventions and programs delivered remotely, remote user methods align with the design process of such programs. The benefits of a wider recruitment pool and efficiency gains, such as reduced travel time or inconvenience, were expected from past studies on remote research methods [15,20,57]. In past research, these gains are often contrasted against other shortcomings of being remote [58], such as lack of contextual insight, connection problems, audio or video problems, low digital literacy, and the like. Emerging from the technological leap through the COVID-19 pandemic, these shortcomings are diminishing, while the perception of benefits for researchers and participants is increasing. The research teams’ initial reservations were that remote research would be complex for potentially vulnerable user participants and could increase stress or fatigue [59]. There were also reservations that remote participation would not provide rich enough user data; however, in the cases presented here, it was found that this method did provide contextualized insights and increased ecological relevance.

### Implications

This study provides insight into the broader learnings from adapting to remote research practices during the COVID-19 pandemic and beyond. From the findings, we have extracted 4 significant implications for future research and practice.

#### Potential for More Agile Research

Remote research practices may come closer to the ideal of an agile approach to testing (“microtesting”), involving briefer and more frequent evaluation sessions with users. This has also been recommended by other recent publications within mHealth [3,60]. For researchers to take advantage of this potential for mHealth apps and interventions, it will bring mHealth research closer to agile user experience practice [61-64] and continuous testing of minimum viable products or prototypes as a form of hypothesis testing [65]. Remote methods facilitate fast cycle iterations and testing in a research design process of sensemaking through trial and error [66].

#### Remote Research Increases Ecologic Relevance

The interventions developed in these research programs were designed to be used within the context of users’ lives, usually the home. Thus, a remote testing method was more ecologically relevant than a traditional face-to-face user test in an office setting. We evaluated the interventions using remote methods in the user’s home and on their devices. This enables contextual inquiry and enhances the representativeness of research findings and the applicability of the designed solution. Screen sharing from a mobile device has also improved [67] compared with during our data collection; this will reduce the problems of remote testing of mHealth interventions, enabling testing and feedback sessions with users in their own contexts and on their devices with direct interaction on the app [68].

#### Technology Impacts Privacy and Human Action

Our research found that there is a risk of capturing more personal data than planned through the ease of recording and screen sharing when engaging with participants through web-based modes. As the participant joins from home, their home context is recorded, including background information and activity. Screen sharing from the participant’s device may enable accidental capturing of on-screen activity, such as open tabs and browsing history, which may be unintentional on the participant’s part. Digital ethnographers have highlighted this factor in previous studies [69]. This highlights the need to safeguard participants’ privacy, as participants may not fully grasp the need to protect their privacy [70]. Throughout the research, participants became more aware of how to protect their privacy, which is represented by the increasing use of video filters such as blurred backgrounds, muting cameras, or strategically placing the webcam. However, some participants perhaps showed unintended details of their personal lives. Researchers should be aware of the ethical considerations of recording videos of participants in their home environment and take care to protect their privacy. Consenting protocols, which include preparing participants for digital interactions, are essential so that participants are adequately informed and aware. As researchers, we may also incorporate practices from web-based counseling and telehealth. Researchers in telehealth also call for revisiting ethical guidelines and procedures following the “ongoing natural experiment” of the pandemic [71].

Our research suggests that when selecting technologies for remote research, it is necessary to consider their functionality regarding privacy protection and the mediating role of technology [58] on human action [72,73]. For instance, when we choose Zoom, Discord, or any other technology, we should consider the norms of how these technologies are being used in other contexts, how these patterns might influence researchers and participants, and how this may influence the data collected.

#### Remote Research Leads to User Involvement on Participants’ Terms

Researchers were concerned by the limited opportunity that remote methods present for informal conversation and rapport building. This interaction style enables trust building and may make research participation more comfortable and less intimidating. However, we found that remote methods shifted control to participants and offered greater comfort than attending unfamiliar institutional settings for face-to-face sessions. Remote methods have the potential for enhanced anonymity as participants have more control over what they share. This may be particularly pertinent for research that involves sensitive or taboo topics, allowing individuals to feel more at ease sharing their experiences and perspectives [74,75].

It was our experience, during work-from-home COVID-19 mandates, that power imbalances were diminished as both researchers and participants were dialing in from a home setting. Thus, there was a more equal grounding and reduced power differential [76]. This is worth considering for future research, specifically setting up the research so that participants and researchers are in similar settings during interaction. When 2 researchers dial in from the same physical location, that introduces a new imbalance, and future research should consider applying the principle of “one remote, all remote” [77,78] when there is a need to do hybrid remote research to ensure equal participation.

### Limitations

For the cases in this study, participants were involved in design processes to capture their experience with iterative designs and provide feedback on design revisions. This took place at different time points during the pandemic. The original research was not designed to answer the research questions of this study. Instead, this topic *emerged* [28] through practice and through reflecting on practice [26]. Retrospective studies have limitations since they may depend on a review of data not planned for research use [79], and information may be missing. This has been mitigated by the participation of the 2 lead authors who conducted the original research. However, our interpretation may be biased despite taking a critical stance on our reflections and interpretations.

The cases and findings presented spark conceptual development and analytical discussion [80] on remote user design methods. However, there are also limitations regarding participants and to whom the findings are relevant. Across cases, specific inclusion criteria and requirements related to recruitment likely impacted our ability to recruit participants. For instance, in cases 1 and 2, we could not advertise for participants and relied on third parties to share information about the research project with potential participants. There was also a requirement to speak Norwegian fluently due to the in-app language. These factors may have reduced the number of people with minority or immigrant backgrounds who registered for the research in the Norway project. Bearing in mind that the young unemployed are twice as likely as other young people to have come to Norway as migrants, this is a weakness. Both projects called for narrow recruitment strategies to target specific population groups. Findings from this study reflect the experiences of the population groups that were involved and may not be generalizable. Further research should explore the applicability and benefit of remote user methods across other population groups.

### Conclusions

The COVID-19 pandemic has reshaped the research landscape in many ways, driving rapid innovation and the adoption of remote research methods. These methods proved crucial in overcoming recruitment challenges and enabling researchers to engage with diverse participant groups across geographical areas. Applying remote methods within hard-to-reach groups reduced participation barriers, facilitated recruitment, and cultivated a more inclusive and comfortable research environment. As researchers and designers navigate the evolving research landscape, the lessons learned underscore the enduring value of remote research methods in promoting user participation in the design of mHealth interventions. Furthermore, they may serve as a reminder to question persistent assumptions about technological competence and access in vulnerable populations.

## Abbreviations

CL-APP: Career Learning App
HCD: human-centered design
mHealth: mobile health
NAV: Norwegian Labour and Welfare Administration
REDCap: Research Electronic Data Capture
RFiTT: Responsive Feeding in Tough Times
